# *UNC13B* Promote Arsenic Trioxide Resistance in Chronic Lymphoid Leukemia Through Mitochondria Quality Control

**DOI:** 10.3389/fonc.2022.920999

**Published:** 2022-05-30

**Authors:** Xiao-Bo Wang, Li-Hua Yuan, Le-Ping Yan, Yong-Bin Ye, Bo Lu, Xiaojun Xu

**Affiliations:** ^1^ Department of Hematology, The Seventh Affiliated Hospital, Sun Yat-Sen University, Shenzhen, China; ^2^ Department of Pediatric Surgery, University of Hong Kong-Shenzhen Hospital, Shenzhen, China; ^3^ Scientific Research Center, The Seventh Affiliated Hospital, Sun Yat-Sen University, Shenzhen, China; ^4^ Department of Hematology, Zhongshan Hospital Affiliated to Sun Yat-Sen University, Zhongshan, China

**Keywords:** arsenic trioxide, drug resistance, *UNC13B*, mitochondria, chronic myeloid leukemia

## Abstract

In clinical practice, arsenic trioxide can be used to treat a subset of R/R CML patients, but resistance tends to reappear quickly. We designed an experiment to study arsenic trioxide resistance in K-562 cells. Previously, we identified the *UNC13B* gene as potentially responsible for arsenic trioxide resistance in K-562 cells *via* gene chip screening followed by high-content screening. We aimed to investigate the role and mechanism of the *UNC13B* gene in K-562 cells, an arsenic trioxide-resistant chronic myeloid leukemia cell line. *In vitro* lentiviral vector-mediated *UNC13B* siRNA transfection was performed on K-562 cells. The roles of *UNC13B* in cell proliferation, apoptosis and cell cycle pathways, and colony formation were analyzed by CCK-8 assay, fluorescence-activated cell sorting, and soft agar culture, respectively. Gene chip screening was used to define the possible downstream pathways of *UNC13B*. Western blot was performed to further validate the possible genes mediated by *UNC13B* for arsenic trioxide resistance in patients with chronic myeloid leukemia. *UNC13B* downregulation significantly inhibited growth, promoted apoptosis, decreased colony formation, reduced the duration of the G1 phase, and increased the duration of the S phase of K-562 cells. Western blot results confirmed that *UNC13B* may modulate the apoptosis and proliferation of arsenic trioxide-resistant chronic myeloid leukemia cells through the mediation of MAP3K7, CDK4, and PINK1. *UNC13B* is a potential therapeutic target for patients with arsenic trioxide-resistant chronic myeloid leukemia.

## Introduction

Chronic myeloid leukemia (CML) is a malignant tumor resulting from a clonal proliferation of bone marrow hematopoietic stem cells, accounting for 15% of adult leukemias (1). According to clinical trials, an estimated 20–30% of patients fail to achieve an expected therapeutic effect with the use of tyrosine kinase inhibitors (TKIs) or develop resistance to TKIs after preliminary efficacy is obtained (1). Furthermore, most patients with CML have a high probability of developing blast crisis (2). New-generation TKIs have an increased potency in the treatment of CML; however, the use of these agents fails to prevent disease progression in some patients (3). TKI in combination with X regimen is an option for patients with CML; X drugs include interferon, cytarabine, homoharringtonine, and arsenic trioxide (ATO) (4).

ATO use has been approved for the treatment of patients with primary, relapsed, and refractory acute promyelocytic leukemia. Wang et al. reported that ATO inhibits the Wnt/β-catenin signaling pathway by downregulating the expression of CD44 on the surface of K-562 cells, which ultimately inhibits cell proliferation and prevents tumorigenesis (5). ATO resistance decreases treatment efficacy in patients with leukemia.

To explore the potential mechanism of ATO resistance, we previously developed an ATO-resistant CML cell line (K-562) cells; moreover, we identified that *UNC13B* was highly expressed in such cells *via* gene chip screening. We hypothesized that this gene may be related to ATO resistance in K-562 cells.

## Materials and Methods

### Target Gene Screening

Previously, we identified ATO-resistant K-562 cells (ATCC, Virginia, USA) by culturing K-562 cells with varied concentrations of ATO. The surviving cells were passaged and repeatedly treated with ATO until a stable ATO-resistant cell line was achieved. To evaluate the effect of the target gene on ATO resistance, we isolated and analyzed ATO-resistant K-562 cell RNAs using GeneChip PrimeView Human Gene Expression Assay (Thermo Fisher Scientific, Waltham, MA). From hundreds of highly expressed genes, 20 genes were selected and downregulated for high-content screening *via* the CCK-8 assay. *UNC13B* was identified as a target gene for further analysis in the current study.

### Cell Culture and Transfection

K-562 cells were cultured in an RPMI 1640 medium containing 15% fetal bovine serum at 37°C and an incubator atmosphere of 5% CO_2_. Based on the nucleotide sequence of *UNC13B* in the GenBank database (GenBank: NM 020313.2), the ATO-resistant K-562 cells were transfected with *UNC13B* shRNA (shUNC13B) using a lentivirus vector. Cells were also transfected with non-target shRNA as control (shCtrl). Gene expression was screened 24 h after transfection.

### Reverse Transcription-PCR

Reverse Transcription-PCR was used to analyze *UNC13B* expression levels of the transfected cells after *UNC13B* knockdown, following a previously reported procedure (6). The total RNA was extracted *via* the TRIzol method (Invitrogen, Carlsbad, CA) and reverse-transcribed into cDNA. The amplification reaction was performed with an ABI 7500 system (Thermo Fisher Scientific). GAPDH was used as an internal reference. Some samples were processed according to the instruction of the 3’ IVT Plus kit (Thermo Fisher Scientific) for the evaluation of the genes on a chip.

### CCK-8 Assay

After various treatments, cells were cultured in a 96-well plate for 24, 48, and 72 h. At each time point, 10 µL of CCK-8 was added into the wells and cultured with the cells in the incubator for 4 h. Finally, the optical density at 490 nm was determined *via* a microplate reader (iMARK; Bio-Rad, Hercules, CA).

### Flow Cytometry

Apoptosis and cell cycle analysis were performed by flow cytometry. For cell apoptosis evaluation, annexin-APC was used to stain the cells prior to the test according to the manufacturer’s instruction (BD Biosciences, Franklin Lakes, NJ). Propidium iodide was used to stain the cells for cell cycle examination. The cells were analyzed in a BD FACSCelesta flow cytometer (BD Biosciences). Fluorescence-activated cell sorting was performed to analyze target cell ratios.

### Colony Formation Assay

K-562 cells were collected and suspended with a density of 500 cells/mL using RPMI 1640 medium with 20% fetal bovine serum. We used 9 mL of suspended cells and 1 mL of 3% low-melting-point agarose solution to prepare a cell agarose suspension, which was seeded into a 6-well plate with 3 mL into each well in triplicate. The cells were placed in a refrigerator at 4°C for 10 min and transferred to a cell incubator for culture. After 18 days of culture, the cells were stained with a 0.05% crystal violet staining solution and then observed under a microscope.

### Ingenuity Pathway Analysis

The RNA of the *UNC13B* knockdown ATO-resistant K-562 cells was tested using Ingenuity pathway analysis (IPA; Qiagen, Hilden, Germany) and the 3’ IVT Plus kit (Thermo Fisher Scientific) for downstream gene screening. A gene interaction network was built with IPA, and genes related with the growth and apoptosis of tumor cells were identified; some of these genes were selected for Western blot analysis.

### Western Blotting

The cells were collected and disrupted by sonication, total protein was extracted, protein concentrations were determined using a bicinchoninic acid quantitative kit (Beyotime Biotechnology, China), and sodium dodecyl sulfate polyacrylamide gel electrophoresis was performed. The proteins were transferred to polyvinylidene fluoride (PVDF) membranes and blocked with 5% non-fat dry milk at 4°C for 6 h. Mouse anti-human UNC13B monoclonal antibody (ab97664, abcam) was added to the membrane and incubated overnight at 4°C. Mouse or goat anti-human IgG secondary antibody was then incubated for 2 h. The contents of UNC13B, MAP3K7 (ab109526, abcam), CDK4 (#12790, CST), and PINK1 (ab23707, abcam) proteins on PVDF membranes were detected by chemiluminescence.

### Statistical Analysis

Data are presented as mean ± standard deviation. Student’s t-test was used to assess the differences between two groups. Statistical significance was established at *p* < 0.05.

## Results

### UNC13B Was Positively Correlated With K-562 Cell Proliferation

**Figure 1:**
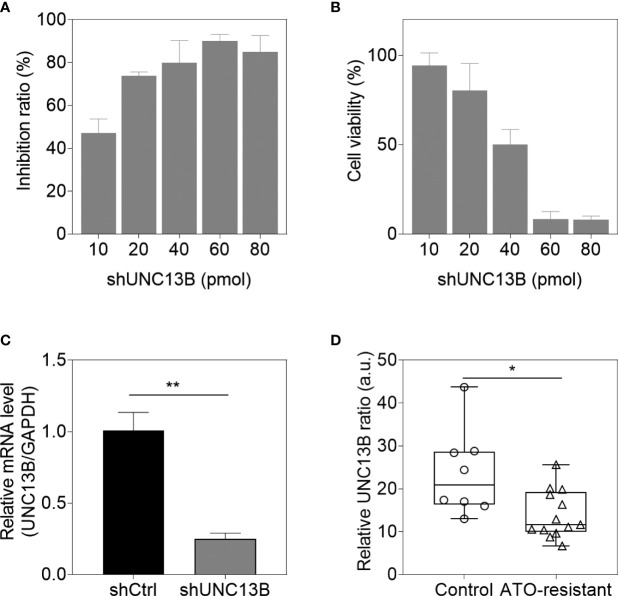
*UNC13B* expression of arsenic trioxide (ATO)-resistant K-562 cells after *UNC13B* knockdown. Cell viability **(A)** and transfection efficiency **(B)** were identified 48 h after transfection in the range of 10 pmol to 80 pmol shUNC13B. **(C)** The cells were analyzed 3 days post-knockdown *via* quantitative PCR. **(D)** Transcriptional levels of UNC13B were identified in ATO-tolerance clinical samples (n=13), control group referred to paracancer tissues (n=8). *p < 0.05, **p < 0.01.

**Figure 2:**
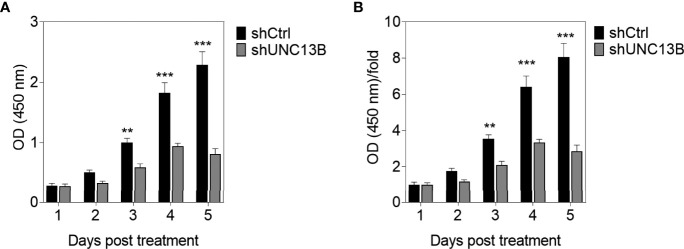
CCK-8 evaluation of the influence of *UNC13B* knockdown on the proliferation of arsenic trioxide (ATO)-resistant K-562 cells. **(A)** Optical density (OD) value of the cells measured at 450 nm and tested *via* CCK-8 assay in different timepoints. **(B)** OD/fold of **(A)**. **p < 0.01, ***p < 0.001.

*UNC13B* (hMUNC13, MUNC13-2, UNC13, Unc13h2) codes UNC-13 homolog B (UNC13B), was a target of diacylglycerol second messenger pathway. UNC13B is primarily located in vesicles, and promotes exocytosis by promoting vesicle maturation. Since our pervious results showed significantly elevated levels of UNC13B in ATO-tolerant cell lines, we first investigated the relationship between UNC13B and cell survival in this study. We transfected UNC13B shRNA (shUNC13B) with a lentivirus vector to analyze the association between UNC13B and cell proliferation. After shUNC13B transfection, *UNC13B* expression in the K-562 cells significantly decreased compared to that of the control (**Figures 1A–C**). UNC13B levels were further identified in clinical samples with ATO-resistant. The results showed that UNC13B levels were significantly increased in ATO-resistant sample compared to para-cancer tissues (**Figure 1D**). CCK-8 analysis showed that *UNC13B* knockdown had a greater inhibitory effect on the growth of ATO-resistant K-562 cells than that of control cells during the 5-day culture period (**Figure 2**).

### UNC13B Downregulation Promotes Cell Apoptosis

**Figure 3:**
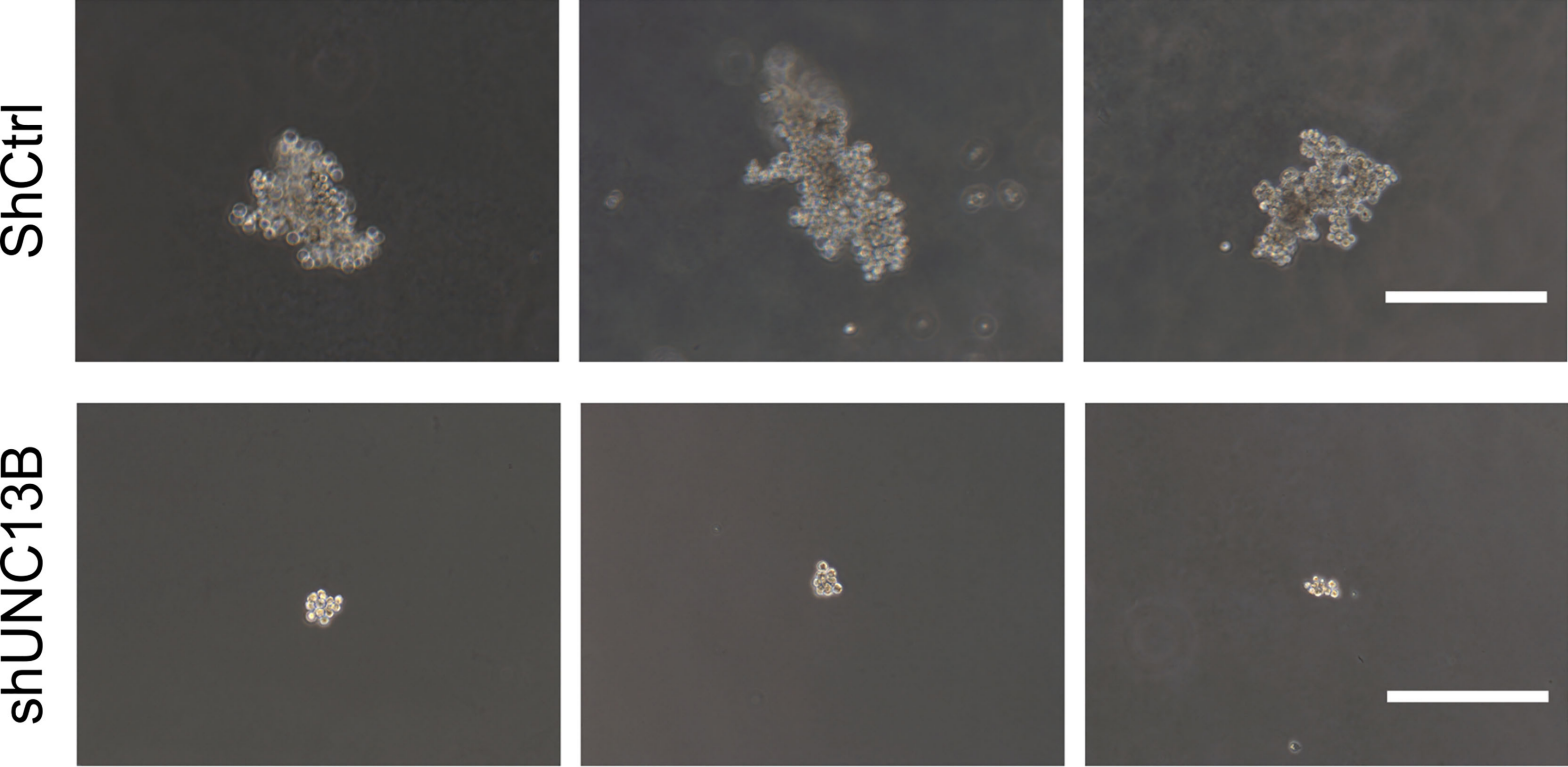
Soft agarose colony formation of arsenic trioxide (ATO)-resistant K-562 cells after *UNC13B* knockdown. Three representative images of each group. Scale bar: 500 µm.

**Figure 4:**
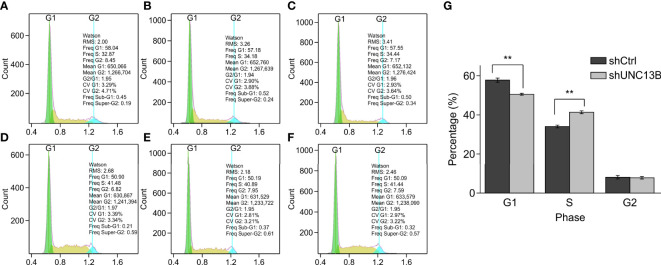
Cell cycle analysis by fluorescence-activated cell sorting (FACS) of arsenic trioxide (ATO)-resistant K-562 cells after *UNC13B* knockdown. **(A–C)** FACS analysis of the shCtrl group. **(D–F)** FACS analysis of the shUNC13B group. **(G)** Quantification of the G1, S, and G2 phases of the shCtrl and shUNC13B groups. **p < 0.01.

Further, we analyzed the reasons for the increase in live cell number, verified the apoptosis, viability and cell cycle after transfection. Flow cytometry analysis indicated a significant increase in the apoptosis ratio of ATO-resistant K-562 cells after *UNC13B* knockdown. Furthermore, the colony formation assay showed that the size of the formed tumors in the shUNC13B group was much smaller than those in the control group (**Figure 3**). Moreover, flow cytometry analysis showed a decrease and an increase in the number of G1 and S phase cells, respectively, among the ATO-resistant K-562 cells, albeit no significant change in the number of G2/M phase cells (**Figure 4**). These results suggest that the down-regulation of UNC13B leads to apoptosis and affects cell viability.

### UNC13B Is Involved in Mitochondrial Fusion Regulation

**Figure 5:**
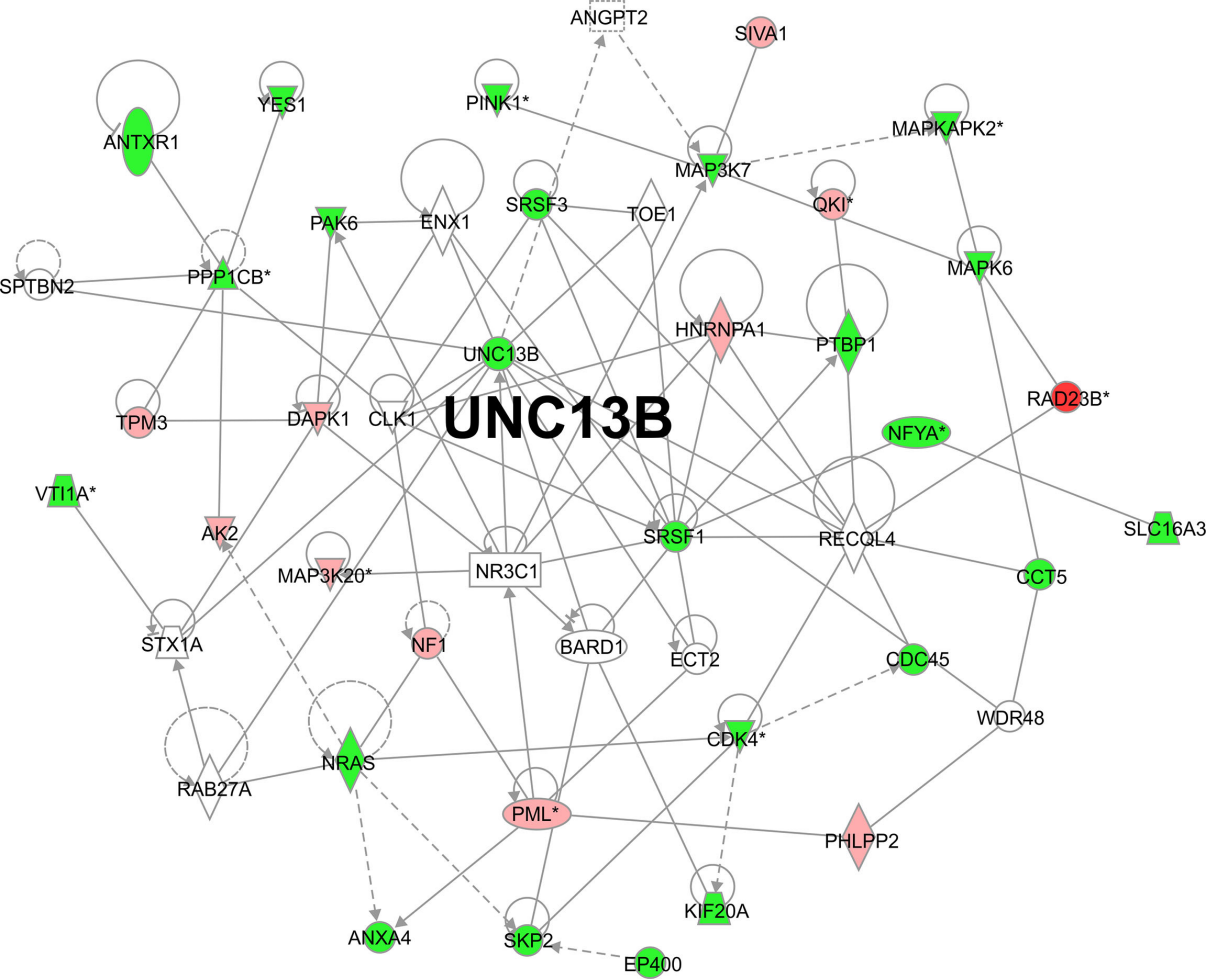
Gene interaction network based on ingenuity pathway analysis. *p<0.05.

**Figure 6:**
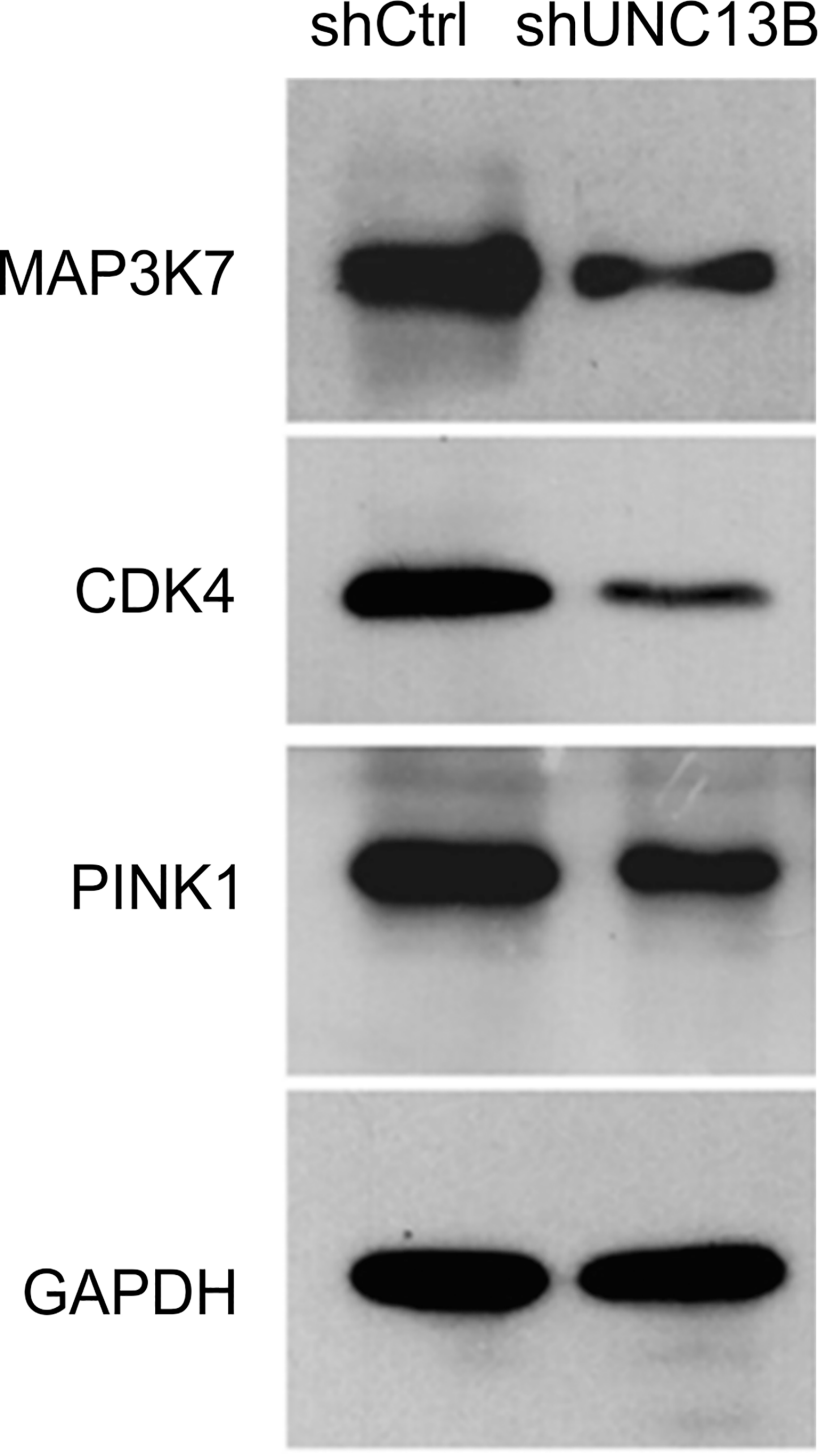
Western blotting analysis of key proteins mediated by *UNC13B*.

We analyzed downstream genes involved in UNC13B, in the gene interaction network, approximately 20 genes showed reduced expression after *UNC13B* knockdown (**Figure 5**). IPA demonstrated that *UNC13B* may affect downstream genes and the proliferation and apoptosis of K-562 cells through an interaction with NRAS and SRSF1, genes with significant differences included PINK1 (mitochondrial fusion), PPPICB (chromosome structure, cell cycle),QKI (RNA binding protein), MAPKAPK2 (DNA damage), NFYA (DNA binding), MAP3K20 (cell cycle, signaling), CDK4 (cell cycle G1 phase), PML (apoptosis), VTI1A (protein transport) (**Figure 5**). According to the results of **Figure 4**, we excluded genes related to cell cycle, we further evaluated genes *via* western blot and found that *UNC13B* knockdown induced an obvious upregulation of MAP3K7, CDK4, and PINK1 proteins (**Figure 6**). Above results indicated that UNC13B regulated both apoptosis and mitochondrial fusion, and further promote cell tolerance to ATO.

## Discussion

Most patients with CML improve considerably with TKI treatment; however, CML cannot be cured using TKI (7), because TKIs cannot completely eliminate leukemia stem cells from the bone marrow, which is the root cause of relapse in patients with chronic-phase CML after remission. After more than 20 years of observing the effects of TKIs, it has been clinically found that TKIs do not have a good therapeutic effect on patients with CML in the accelerated phase or during blast crisis. Moreover, some patients respond slowly to TKI treatment, whereas others exhibit primary drug resistance. Studies have shown that the abovementioned phenomenon may result from tumor-related gene mutations (8), novel *BCR-ABL* transcripts (9), *BCR-ABL* fusion mutations (such as T315I) (10), and long-term medication history (11). Hematopoietic stem cell transplantation (HSCT) remains the only radical cure for CML; nonetheless, based on the relatively slow progress of CML and the relatively high risk of allograft HSCT (allo-HSCT), allo-HSCT is not recommended as the first choice for CML treatment. TKIs are recognized as an early CML treatment method; however, they can only prolong life rather than cure the disease. Once the patient undergoes a sudden change (for example, a T315I mutation), the optimal transplant period is missed, thereby resulting in an extremely poor prognosis.

By the 19^th^ century, Fowler’s solution, or ATO, was used in the treatment of CML. Before the advent of chemotherapeutics, ATO was the gold standard for CML treatment. A 2-week ATO treatment course can achieve complete hematological remission in 74% of patients with chronic-phase CML (12, 13). Due to its unique mechanism of action, ATO plays a unique role in CML treatment, even in the era of TKIs (14). ATO reportedly destroys CML cells by reducing *PML* expression and kills CML tumor stem cells (15). Therefore, ATO has excellent application prospects in the eradication of CML, thereby bringing new treatment opportunities for patients with CML who could not benefit from HSCT. Additionally, two clinical studies have shown that 74% of patients who undergo ATO treatment for CML attain complete remission (12, 13).

ATO has been used in combination with other drugs in a variety of cancers, such as T-cell acute lymphoblastic leukemia (16), myelodysplastic syndromes (17), idiopathic thrombocytopenic purpura (18), acute myeloid leukemia (19), and multiple myeloma (20); moreover, it has been used in patients with both newly diagnosed and refractory or relapsed cancers. ATO shows a certain degree of curative effect, and so our group has been examining ATO resistance in leukemia. In the early stage of the current study, *UNC13B* expression was found to be increased in tumor cells of ATO-resistant patients (data not shown). Through *in vitro* experiments, it was found that *UNC13B* expression was significantly increased in ATO-resistant K-562 cells; hence, we speculate that *UNC13B* is an important gene for ATO resistance.

We found that the growth ability of CML K-562 cells was significantly inhibited after downregulation of *UNC13B*. To clarify the underlying mechanism, we investigated changes in relevant regulatory molecules in K-562 cells.

*NRAS* (neuroblastoma RAS viral oncogene homolog) belongs to the RAS guanosine triphosphatase gene family. Acting as a molecular switch, NRAS is located on the cell membrane surface and can transfer extracellular signals to the nucleus to regulate cell proliferation, differentiation, and apoptosis (21). Patients with acute myeloid leukemia and *NRAS* mutations have a low overall complete remission rate of approximately 10% (22).

SR proteins are responsible for regulating constitutive and alternative splicing in genes and may shuttle between the nucleus and cytoplasm. *SRSF1* (serine/arginine-rich splicing factor 1), a typical SR protein family member, is localized on chromosome 17q23 and contains an N-terminal RRM domain and a C-terminal RS domain. The RRM domain is responsible for mediating RNA binding and determining the substrate specificity of SR proteins. The RS domain is mainly involved in protein-protein interactions and regulates the formation of SRSF1-ESE complexes *via* phosphorylation and dephosphorylation of serine residues. ESE binding to SRSF1 can activate and inhibit splicing. SRSF1 regulates alternative splicing by recognizing and binding to ESEs (23). *SRSF1* is a key target of Myc, which directly or indirectly binds to the *SRSF1* promoter region and activates its transcription (24).

Furthermore, it has been found that dinaciclib, a CDK1/2 inhibitor, produces cytotoxicity by inhibiting the expression of *CHD1* and *MAP3K7* (a recently discovered protein), and prevents the proliferation of mouse prostate cells (25). CDK4 is one of eight cyclin-dependent kinases, all of which can bind to d-type cyclins after mitotic signal activation to form CDK4-cyclin complexes, thereby promoting reverse transcription and playing a role in the process of cell proliferation (26). In tumor cells, CDK4/6 hyperactivation results in genomic and chromosomal instability with uncontrolled cell proliferation, which ultimately leads to abnormal cell cycle regulation (27). PINK1 promotes tumor survival, protects cancer cells from different cytotoxic agents (28), and exerts its biological function through oxidative stress (29). In the present study, we found that the downregulation of *UNC13B* could in turn downregulate MAP3K7, CDK4, and PINK1 in K-562 cells; hence, *UNC13B* might directly or indirectly promote the biological activity of ATO-resistant CML cells by affecting the expression of the abovementioned proteins.

PTEN induced kinase 1(PINK1) is mainly located in mitochondria, and its function is to inhibit mitochondrial dysfunction (30). Mitochondria are the site of oxidative metabolism in eukaryotes, where sugars, fats and amino acids eventually oxidize and release energy. At the same time, mitochondria can also store calcium ions and act synergistically with structures such as endoplasmic reticulum and extracellular matrix to control the dynamic balance of calcium ion concentration in cells.

Previous reports have shown that PINK1 is essential for mitochondrial quality control (30). PINK1 inhibition leads to mitochondrial excessive fusion, and further improve the efficiency of oxidative phosphorylation (31). In our results, the expression level of PINK1 was also significantly decreased with the inhibition of UNC13B. This suggests that the quality control of mitochondria will be significantly affected during UNC13B down-regulation, and the accompanying metabolic disorders will further affect the cell energy metabolism.

Due to insufficient experimental funds, this experiment did not conduct gene chip experiments, nor did it detect the entire series of proteins in major signaling pathways.

We found that *UNC13B* downregulation may in turn downregulate the contents of MAP3K7, CDK4, and PINK1 in K-652 cells. Moreover, *UNC13B* may directly or indirectly promote the biological activities of ATO-resistant CML cells by affecting the abovementioned proteins, the underlying mechanism may involve mitochondrial quality control. These findings provide potential benefits for the treatment of patients with CML. Furthermore, our study may provide a treatment strategy for the clinical treatment of non-ATO-resistant CML. Although the findings of this study are very promising, they should be verified *via* further studies.

## Data Availability Statement

The raw data supporting the conclusions of this article will be made available by the authors, without undue reservation.

## Author Contributions

X-BW performed the experiments, prepared the figures and wrote the manuscript. BL, Y-BY, and L-HY collected and analyzed data. L-PY and XX initiated and directed the whole research. X-BW, L-HY and L-PY contributed to the manuscript equally. All authors contributed to manuscript revision, read, and approved the submitted version.

## Funding

This work was supported by the Zhongshan Science and Technology Research Major Project (No. 2017B1002), Shenzhen Science and Technology Plan Basic Research Project (No. JCYJ20180307150408596; No. JCYJ20190809172403604), Sanming Project of Medicine in Shenzhen (No. SZSM201911004), Starting Grant from The Seventh Affiliated Hospital Sun Yat-sen University (No. ZSQYRSF0008) and Young Teacher Fostering Grant from Sun Yat-sen University (No. 20ykpy18), and Natural Science Foundation of Guangdong Province (No. 2019A1515110703).

## Conflict of Interest

The authors declare that the research was conducted in the absence of any commercial or financial relationships that could be construed as a potential conflict of interest.

## Publisher’s Note

All claims expressed in this article are solely those of the authors and do not necessarily represent those of their affiliated organizations, or those of the publisher, the editors and the reviewers. Any product that may be evaluated in this article, or claim that may be made by its manufacturer, is not guaranteed or endorsed by the publisher.
